# Trends in, and Risk Factors for, Suicide in Public Places: A 17‐Year Case–Control Study in Australia

**DOI:** 10.1111/sltb.70017

**Published:** 2025-04-08

**Authors:** Sangsoo Shin, Matthew J. Spittal, Angela Clapperton, Jane Pirkis, Lay San Too

**Affiliations:** ^1^ Centre for Mental Health and Community Wellbeing, Melbourne School of Population and Global Health The University of Melbourne Parkville Victoria Australia

**Keywords:** Australia, home, public place, suicide

## Abstract

**Objective:**

To examine factors associated with the choice of public location over home to die by suicide.

**Methods:**

This study used a case–control design. Data on suicides that occurred between 2001 and 2017 in Australia were extracted from the National Coronial Information System. Cases were suicides that occurred in public places and controls were suicides that occurred at home. Logistic regression models were used to estimate the associations between suicide location and several sociodemographic variables, depending on whether hotel rooms were included in or excluded from public places.

**Results:**

In total, 25.2% of 42,656 suicides occurred in public places including hotel rooms, 69.3% at home, and 5.4% in nonpublic places other than at home (e.g., inpatient ward or correctional facilities). Excluding suicides in hotel rooms from public places, 1.6% points of suicides in public places moved to nonpublic places other than at home. In multivariable regression models regardless of scenarios, males (compared with females) had higher odds of dying by suicide in public places, while those who were divorced/separated/widowed (compared with married people), those who were older (aged 30–54, and aged 55 and above, compared with under 30 years), and those who were unemployed or not in the labor force (compared with employed people) had lower odds of suicide in public places.

**Conclusion:**

The findings should be used to inform the design of strategies to prevent suicides in public places.

## 1 Introduction

Suicide is recognized as a major public health issue in Australia and globally. Age‐standardized suicide rates have increased in Australia from 11.2 per 100,000 in 2010 to 11.8 in 2023 (ABS 2024), which corresponds 2480 suicide deaths in 2010 and 3214 in 2023. These figures indicate an increase of about 8.0% in the suicide rate during the last decade.

Recently, increased attention has been paid to suicides that occur in public places. Preventing suicides in public places is a priority because some of these places can gain a reputation as so‐called “suicide hotspots,” thereby encouraging further suicides at these locations. Furthermore, there has been an investigation into a broader array of dispersed public sites where suicides occur. These include open railway networks (Too et al. 2015), subway networks (Chung et al. 2016) or hotels within a geographical boundary (Chen et al. 2023; Gemar et al. 2008).

Suicides in public places are also of concern because the suicide act itself may be witnessed by others or others may discover the deceased. This may directly impact these individuals (Giupponi et al. 2019). In addition, the methods that are made available by the nature of these sites (such as jumping from heights or jumping in front of moving vehicles) tend to have a high case fatality rate, which means that suicide attempts at these sites are often lethal (Hawton 2007). Finally, media coverage of suicides at these sites (Hamilton et al. 2011) can have a potential “contagion effect,” reinforcing the notoriety of known “suicide hotspots” and leading to previously unknown sites gaining a new reputation as such.

Although research into suicide in public places has been expanding, understanding of the epidemiology of suicide in public places remains relatively poor. A handful of studies have described the epidemiology of suicide at “suicide hotspots” (Berman et al. 2022; Pirkis et al. 2015; Sinyor et al. 2017), but only a few studies have provided a descriptive picture of suicides in other public places (e.g., Devon county in England (Owens et al. 2009)). Most of these studies have presented descriptive statistics only and not offered any point of comparison. The present study aimed to characterize suicides in the full range of public places at an Australian national level, comparing these with suicides occurring at home.

## 2 Method

### 2.1 Study Data and Design

This study used a case–control design. Data on deaths classified as intentional self‐harm (defined as suicide in this study) that occurred between January 1, 2001, and December 31, 2017, in Australia were obtained from the National Coronial Information System (NCIS). The NCIS compiles sociodemographic and geographical information on deaths reported to coroners in Australia and New Zealand.

### 2.2 Inclusion and Exclusion Criteria

Suicides were classified as occurring in public places, at home, and in nonpublic places other than home. The definition of public places was developed from a previous report (Owens et al. 2015). Suicide locations satisfying at least one of the following conditions were classified as public places: (1) land or water that was open to the public and owned by government or individual(s) (except where the deceased was the owner), (2) any part of the transport or inland waterways network, (3) any accommodation that provided short‐term stays to the public, or (4) all or part of a building that was open to or designed to be used by the public.

This definition was used as the starting point, and all suicides that indisputably occurred in public places were identified on this basis. All suicides that unequivocally occurred at home and in nonpublic places other than home were then identified. This initial classification was done automatically, based on location codes recorded in the NCIS.

This resulted in a residual set of suicides that could not be classified on the basis of the location code alone, and the text in all relevant file attachments (e.g., police narrative of circumstances, autopsy reports) was reviewed to classify them. There were some nuances here. For example, suicides that had a hospital location code were classified as occurring in a public place if they occurred in the hospital car park but in a nonpublic place if they occurred on an inpatient ward. Including these cases, most suicides where the public is not accessible or is not permitted to enter (e.g., military camps, construction sites, workplaces, correctional facilities, and aged care homes) were classified into the category of suicides in nonpublic places other than home.

**FIGURE 1:**
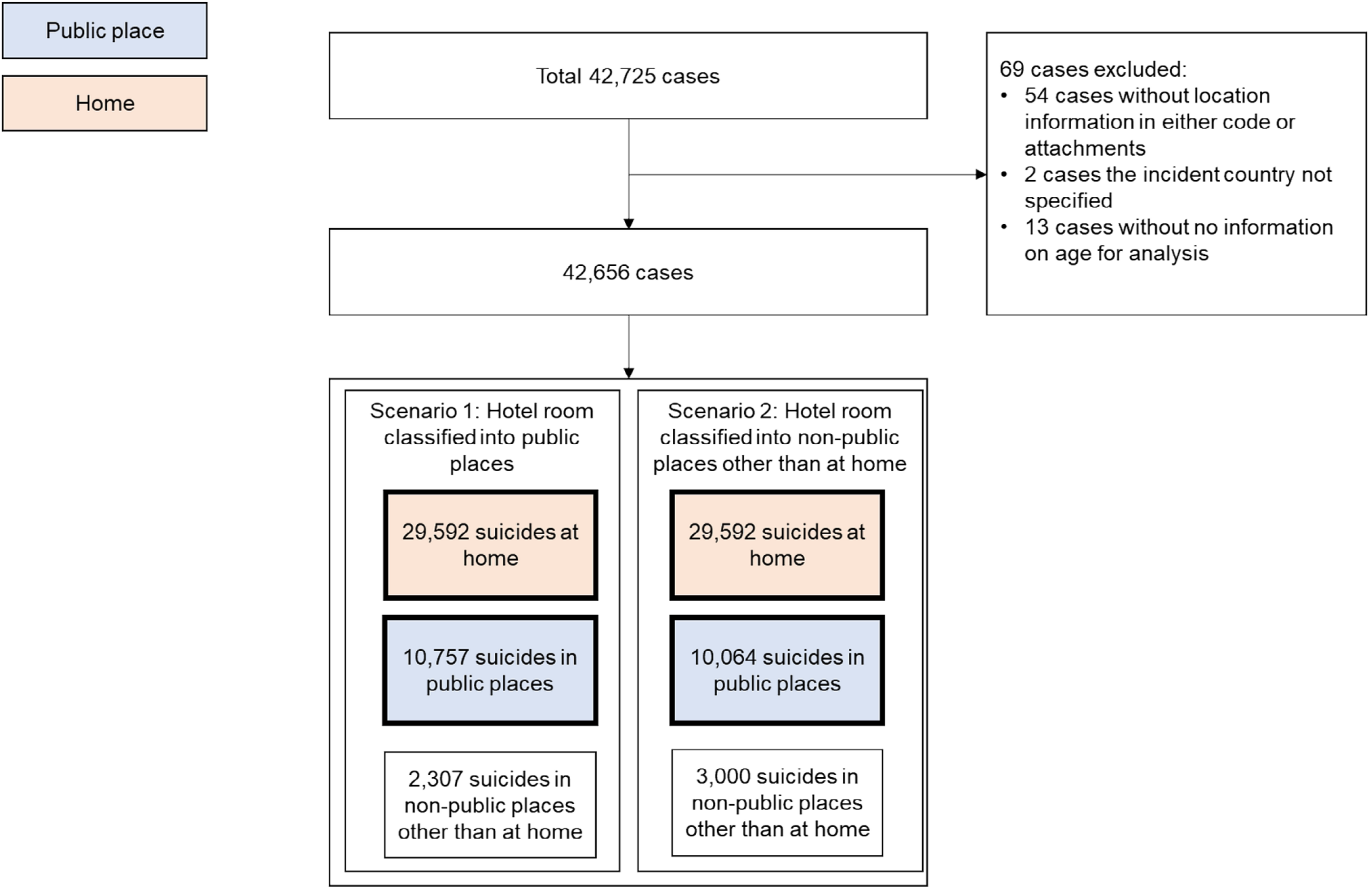
Flowchart of the inclusion procedure of cases upon scenario.

Suicides occurring in hotels were a particular case in point. Suicides occurring in a hotel foyer or other publicly accessible part of the building were automatically classified as suicides in public places. Additional consideration was given to suicides occurring in individual hotel rooms. The abovementioned report classified hotel rooms as public places because they are available for and accessible to the public for booking short‐term stays (Owens et al. 2015). However, access to hotel rooms is restricted if they are occupied by others. For this reason, two scenarios were considered. The first categorized all suicides in hotels as suicides in public places, and the second classified them as suicides in nonpublic places other than at home. In the second scenario, suicides occurring in areas of a hotel accessible to the public (e.g., the pool or rooftop) were still categorized as suicides in public places. Two separate sets of analyses were conducted to reflect the two scenarios (see Figure 1).

In individual situations where a classification could not be made because of insufficient information in the attachments, suicides were classified using the most common classification allocated to other suicides with the same location code. Only cases with no location information in both the coding and the attachments were excluded.

### 2.3 Statistical Analysis

Descriptive analyses were conducted that identified the proportion of suicides by each type of place (public place, home, and nonpublic place other than at home) and profiled the trends for each. Suicides occurring in nonpublic places other than home were then excluded from further analyses. Multivariable logistic regression analyses were then conducted to identify those factors associated with suicide in public places. Suicides occurring in public places were treated as cases and suicides occurring at home as controls. The exposure variables included age (≤ 29, 30–54, ≥ 55), sex (male and female), marital status (married, never married, divorced/separated/widowed, unknown), employment status (employed, unemployed, not in the labor force, and other/unknown), and Indigenous status (non‐Indigenous, Indigenous, and unknown). All statistical analyses were conducted using R 4.40. Note that two sets of descriptive and multivariable analyses were conducted in each case—one in which hotel room suicides were included in suicides among suicides in public places and the other in which they were excluded.

## 3 Results

### 3.1 Proportions of, and Trends in, Suicides in Public Places by Type of Locations

**FIGURE 2:**
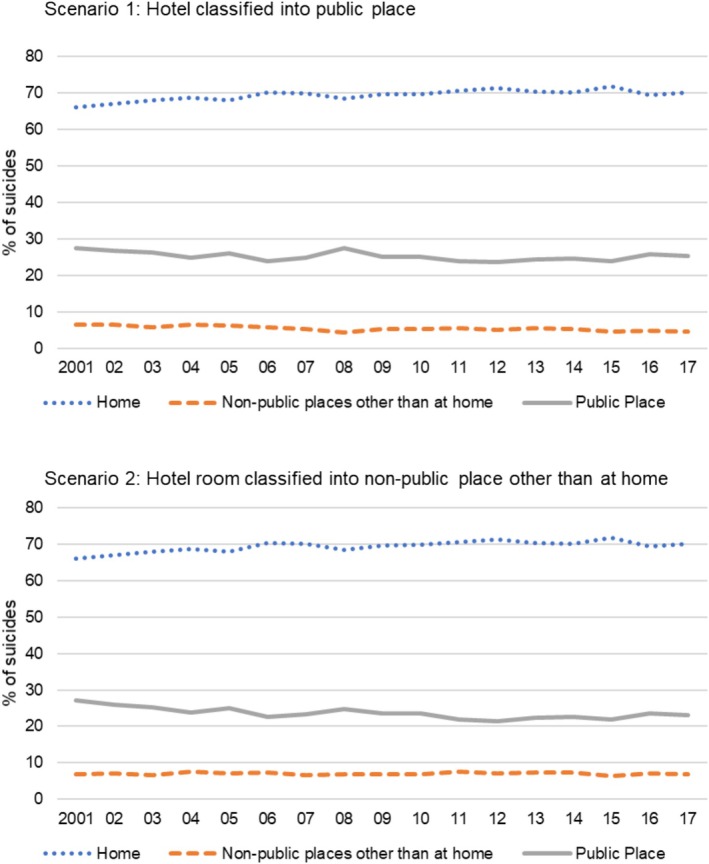
Proportion of suicides by the type of suicide location between 2001 and 2017.

Figure 2 shows that, in total, 42,656 suicides were identified from the NCIS over the 17‐year period. In the first scenario, of these suicides, 25.2% took place in public places (*n* = 10,757), 69% (*n* = 29,592) occurred at home, and 5.4% (*n* = 2307) occurred in nonpublic places other than at home (Figure 2). In the second scenario, 23.6% of suicides show that the proportions of suicides occurring in public places and the suicides in nonpublic places other than at home increased by 7.0%.

### 3.2 Sociodemographic Characteristics of Suicides in Public Places

The sociodemographic characteristics of suicides occurring in public places and the home are shown in Table 1. Suicides in public places, including hotel rooms, were 10,757, and suicides in public places excluding hotel rooms were 10,064. Suicides at home were 29,592.

**TABLE 1:** Characteristics of suicides in public places and suicides at home.

	Public places		Home (*n* = 29,592)
	Public places including hotel room (*n* = 10,757)	Public places excluding hotel rooms (*n* = 10,064)	
Sex			
Females	2110	1888	7383
Males	8647	8176	22,209
Age group			
55 and over	2220	2030	8440
30–54	5660	5255	14,752
15–29	2897	2779	6400
Indigenous status			
Non‐Indigenous	8429	7873	23,961
Indigenous	537	524	1377
Unknown	1791	1667	4254
Marital status			
Married	3461	3235	10,259
Never married	3234	3068	7832
Divorced/separated/widowed	1981	1829	7187
Unknown	2081	1932	4314
Employment status			
Employed	4248	3972	11,140
Unemployed	2422	2268	7077
Not in the labor force	2170	2052	8715
Others/unknown	1917	1772	2660

In both scenarios, regardless of whether hotel suicides were included or excluded from public places, the majority of deaths were among males aged 30–54, Nonindigenous individuals, married persons, or those who were employed. Similarly, for suicides occurring at home, the predominant demographic consisted of males aged 30–54, non‐Indigenous individuals, married persons, or employed individuals.

### 3.3 Predictors of Suicides in Public Places

Table 2 shows the multivariable results from the two models relating to the predictors of suicides in public places (comparing with suicides at home). Model 1 included suicides occurring in hotel rooms in suicides in public places and Model 2 excluded these suicides. In Model 1, males had higher odds of suicide in a public place than females (Odds Ratio [OR] = 1.34; 95% CI = 1.26–1.41). Compared to those aged 29 years and under, those aged between 30 and 54 years and those aged over 55 years were each associated with lower odds of suicide in a public place (OR_30–54_: 0.88, 95% CI = 0.83–0.94, OR_≥ 55_: 0.72, 95% CI = 0.67–0.78). Being divorced/separated/widowed had lower odds of suicide in a public place (OR_divorced/separated/widowed_: 0.83, 95% CI = 0.78–0.89) than those who were married. Being never married was associated with higher odds of suicide in public places over home compared to being married (OR_never married_: 1.12, 95% CI: 1.05–1.19). Those who were unemployed and not being in the labor force had lower odds of suicide in a public place than those who were employed (OR_unemployed_: 0.83, 95% CI = 0.78–0.89, OR_not‐in‐the‐labor‐fource_: 0.76, 95% CI = 0.71–0.81). Indigenous status was not associated with the location of suicide. The results for Model 2 were similar to those for Model 1.

**TABLE 2:** Multivariate logistic regression results for the association between background characteristics and the likelihood of choosing public places over home as a suicide location.

	Model 1 (*n* = 40,349) (Public places including hotel room)		Model 2 (*n* = 39,656) (Public places excluding hotel room)	
	aOR	[95% CI]	aOR	[95% CI]
Total	**0.33**	**[0.30–0.36]**	**0.37**	**[0.35–0.39]**
Sex				
Females*	1.0		1.0	
Males	**1.34**	**[1.26–1.41]**	**1.42**	**[1.34–1.50]**
Age group				
29 and under*	1.0		1.0	
30–54	**0.88**	**[0.83–0.94]**	**0.86**	**[0.81–0.91]**
55 and above	**0.72**	**[0.67–0.78]**	**0.69**	**[0.63–0.74]**
Indigenous status				
Non‐Indigenous*	1.0		1.0	
Indigenous	1.01	[0.91–1.12]	0.96	[0.86–1.07]
Unknown	1.06	[1.00–1.13]	1.02	[0.90–1.15]
Marital status				
Married*	1.0		1.0	
Never married	**1.12**	**[1.05–1.19]**	**1.11**	**[1.04–1.19]**
Divorced/separated/widowed	**0.83**	**[0.78–0.89]**	**0.82**	**[0.77–0.88]**
Unknown	**1.21**	**[1.13–1.30]**	**1.20**	**[1.11–1.28]**
Employment status				
Employed*	1.0		1.0	
Unemployed	**0.87**	**[0.82–0.93]**	**0.87**	**[0.77–0.88]**
Not in the labor force	**0.76**	**[0.71–0.81]**	**0.78**	**[0.73–0.83]**
Others/unknown	**1.87**	**[1.74–2.01]**	**1.86**	**[1.73–2.00]**

*Note:* Significant associations are in bold.

Abbreviations: aOR = adjusted Odds Ratio, CI = Confidence interval.

* Reference category.

## 4 Discussion

Using a nationally representative dataset, the NCIS, the proportion of, and trends in, suicides by type of location were identified. To better understand the trends, the study population was limited to suicides in public places (including and excluding suicides in hotel rooms) and the home only. The associations of various sociodemographic variables with public places versus the home as the suicide incident place were explored using logistic regression in which suicides at home were set as the control group.

The study showed that, in the first scenario categorizing hotels as public places, a quarter of suicides in Australia occurred in public places, 69.3% at home, and 5.4% in nonpublic places other than home. In the first scenario, considering hotel rooms as public places other than home, suicides in this category slightly increased by 7.0%. The absence of comprehensive studies in descriptive epidemiology with nationally representative data, along with varying definitions of public place, impedes straightforward cross‐country comparisons. Given the indirect comparison between studies providing descriptive information on the proportion of suicides that occur in the home, the proportion of suicides occurring in public places in Australia appears to be relatively lower than those in other countries. For example, while 69.4% of suicides in this current study occurred at home, 58.2% of suicides were reported at home in Hong Kong (Yeung et al. 2022); 65.8% in Belgium; 64.7% in France; and 62.1% in New Zealand (Rhee et al. 2016).

The proportion of suicides occurring in public places remained steady over the study period despite the fact that there has been a substantial decrease in suicides by particular methods that used to be common in public places. In Australia, suicides by motor vehicle exhaust gas, a proportion of which likely occurred in public places, decreased substantially during the early 2000s, from 498 in 2001 to 231 in 2006 (Studdert et al. 2010) and have decreased further to 150 in 2017 (Burnett et al. 2021). In 1998, Australia introduced regulations that all vehicles sold have a maximum carbon monoxide exhaust emission level of 2.1 g/km from the previous level of 9.6 g/km (Brennan et al. 2006). The change in carbon monoxide emission standards results in less lethality of MVEG suicide attempts (Spittal et al. 2012). Also, several restrictions on access to suicide methods introduced to public places are known to have been effective (e.g., barriers on a bridge (Law et al. 2014), removal of railway level crossings (Clapperton et al. 2022)). The fact that the proportion of suicides occurring in public places is constant over time despite meaningful drops in suicides by site‐specific suicide methods available in public places implies that the increase in suicides by other methods might offset the decrease by means of restriction. This suggests the need for an analysis of which suicide methods become frequent in public places.

The results suggested that some sociodemographic factors may increase the likelihood of suicide occurring in public places versus the home. Males were more likely to choose public places over the home in comparison with females. This result is, in part, consistent with a previous study conducted in a county of the United States, where females were more likely to choose individual private residences for suicide whereas males chose outdoor locations (Kposowa and McElvain 2006). Several suicide methods known to be lethal are site‐specific; for example, jumping from a high place and jumping in front of a moving object such as a train or car. These violent methods are more common among males (Bennewith et al. 2007; Perron et al. 2013; Taylor et al. 2016). Choosing to attempt suicide in a public place using methods readily available at home (e.g., hanging, poisoning by drug) might be one way to decrease the likelihood that family or another close person would interrupt the suicide attempt, which in turn could lead to higher method‐specific case fatality. In fact, hotels appear to be a location that is more common among males (Chen et al. 2023). Our result, a relatively greater odds ratio for males in Model 2 compared to Model 1, might align with the previous research.

Compared with the younger age group under 30, the middle and older age groups were less likely to choose the public place over home. This phenomenon is possibly related to the tendency to choose more lethal suicide methods in young people. Age itself is a risk factor for case fatality across and within suicide methods because the physiological weakening of those elderly age groups probably accounts for part of this effect (Elnour and Harrison 2008). Therefore, the elderly group might be less motivated to look for suicide methods with higher fatality rates available outside home. They might choose the suicide method to complete their act at once, considering their physical and mental condition or even the possibility of abandoning intervention opportunities.

Marital status also predicted the odds of choosing a public place over the home. Family or cohabitant is not only an asset for functioning suicide prevention by providing familial support (Prabhu et al. 2010), but also could be the last person to intervene with the person in crisis at the very last moment of a suicide attempt. Consistent with this interpretation, in the current study compared to married individuals, those who were divorced/separated/widowed were less likely to choose a public place than the home. In Australia, 33.2% of people who are separated/divorced and 54.3% of those who are widowed live alone, whereas only 0.7% of those who are married and 17.3% of those who have never been married live alone (De Vaus and Qu 2015). This could imply that individuals who were divorced, separated, or widowed had a reduced likelihood of encountering interruption by another person when attempting to end their lives at home. Meanwhile, the result also found people who are never married had higher odds of choosing public places than home as a location. 77.5% of people who have never married live with someone in Australia (De Vaus and Qu 2015). Similarly, as described above, they might decide on another place than home to avoid intervention by others.

Those who were unemployed or not in the labor force were less likely to choose a public place over the home when compared with those who were employed. It is possible that a person being employed might expose them to various locations while commuting, to economic activity, and even to social activity relative to people who are unemployed or not in the labor force. Therefore, employed people might have more information on places other than the home where they could attempt suicide, and they could engage in suicidal action when in the middle of economic or social activities. However, this explanation is speculative, particularly because the association between the other/unknown employment status category with the outcome was stronger and in the opposite direction to the associations with the other employment categories.

Most of the research into interventions to prevent suicide in public places has been concerned with preventative actions that might be taken at so‐called “suicide hotspots”. Much of this has examined the effectiveness of restricting access to means at these sites (e.g., by installing barriers), and has shown that these measures not only prevent suicide at the sites in question (Pirkis et al. 2015) but usually do not lead to significant displacement to nearby sites where the same suicide method is available (Berman et al. 2022; Law et al. 2014). There are other interventions too, although less work has been done on examining their effectiveness and the evidence is not as strong. One of these is a set of interventions designed to capitalize on the fact that others are likely to be present at these sites and other public places. These involve increasing the likelihood that a third party will intervene if someone is showing signs of distress (e.g., bystander interventions, training of site staff, installation of CCTV cameras) (Pirkis et al. 2015; Ross et al. 2020). Another set of interventions relates to encouraging help‐seeking on the part of the person who is at risk (e.g., signs or broader campaigns promoting details of local crisis lines) (King and Frost 2005; O'Neill et al. 2021).

Overall, the two models showed a similar magnitude of associations between variables. This might suggest that hotel room suicides share characteristics with suicides in public places. However, it is possible that the lack of statistical power due to the small number of suicides in hotel rooms might fail to identify meaningful differences in their characteristics.

This is the first study to examine the trends of suicides in Australia by location type and the first case–control study to identify predictors of suicides occurring in public places rather than the home using a nationally representative dataset. However, despite these strengths, this study has several limitations. First, there is a possibility of misclassification of incident place due to coding error by either the researcher or data manager or by changes to coding guidelines over the study period. To minimize data loss and the possibility of misclassification, all available documents for cases with an unclear location type based on coded information on the NCIS were reviewed. Second, it was not possible to examine some important variables such as mental health status and other stressors that were not coded on the NCIS. A more comprehensive understanding of the risk factors for suicides in public places is required to inform the design of interventions to prevent suicide in public places. Finally, it is worth acknowledging that the outcomes could potentially suffer from the underreporting of suicides due to ongoing coronial investigations at the time of the analysis. To address the first concern, measures have been taken to mitigate the impact by selecting a timeframe during which the proportion of closed cases exceeds 95%, from 2001 to 2017. Furthermore, the presence of unknown cases in variables such as marital status, employment status, and indigenous status could potentially introduce bias and influence the results.

## 5 Conclusions

Preventing suicides in public places is imperative in part because of the effect it can have on the public. To date, the general prevention focus has been mostly limited to restricting access to means and discussion of its effectiveness. This study found that the proportion of suicides occurring in public places remains constant despite significant efforts to reduce suicides in public places, focused on means restriction. Therefore, the findings from this study should be used to inform the development of broader strategies to prevent suicides in public places.

## Author Contributions

**Sangsoo Shin:** conceptualization (lead), data curation (lead), formal analysis (lead), methodology (lead), writing – original draft (lead), writing – review and editing (lead). **Jane Pirkis:** conceptualization (supporting), supervision (supporting), writing – original draft (supporting), writing – review and editing (supporting). **Angela Clapperton:** conceptualization (supporting), methodology (supporting), writing – original draft (supporting), writing – review and editing (supporting). **Matthew J. Spittal:** formal analysis (supporting), methodology (supporting), writing – original draft (supporting), writing – review and editing (supporting). **Lay San Too:** conceptualization (lead), data curation (supporting), methodology (lead), supervision (lead), writing – original draft (supporting), writing – review and editing (supporting).

## Ethics Statement

Approval to conduct the study was obtained from the Victorian Department of Justice and Community Safety (project number: M0314) as well as the Human Research Ethics.

## Conflicts of Interest

The authors declare no conflicts of interest.

## Data Availability

The data that support the findings of this study are available on request from the corresponding author, SSS. The data are not publicly available due to it contained information that could compromise the privacy of suicide deceased.
